# Orexins as Novel Therapeutic Targets in Inflammatory and Neurodegenerative Diseases

**DOI:** 10.3389/fendo.2019.00709

**Published:** 2019-10-22

**Authors:** Alain Couvineau, Thierry Voisin, Pascal Nicole, Valérie Gratio, Catalina Abad, Yossan-Var Tan

**Affiliations:** ^1^INSERM UMR1149/Inflammation Research Center (CRI), Team “From Inflammation to Cancer in Digestive Diseases” Labeled by “la Ligue Nationale Contre le Cancer”, University of Paris, Paris, France; ^2^University of Rouen Normandy, INSERM U1234 PANTHER, IRIB, Rouen, France

**Keywords:** orexins, neuropeptides, GPCR, inflammation, neuroprotection, gastroenterology, autoimmune diseases, cancer

## Abstract

Orexins [orexin-A (OXA) and orexin-B (OXB)] are two isoforms of neuropeptides produced by the hypothalamus. The main biological actions of orexins, focused on the central nervous system, are to control the sleep/wake process, appetite and feeding, energy homeostasis, drug addiction, and cognitive processes. These effects are mediated by two G protein-coupled receptor (GPCR) subtypes named OX1R and OX2R. In accordance with the synergic and dynamic relationship between the nervous and immune systems, orexins also have neuroprotective and immuno-regulatory (i.e., anti-inflammatory) properties. The present review gathers recent data demonstrating that orexins may have a therapeutic potential in several pathologies with an immune component including multiple sclerosis, Alzheimer's disease, narcolepsy, obesity, intestinal bowel diseases, septic shock, and cancers.

## Introduction

The G protein-coupled receptors (GPCR) constitute the largest family of membrane receptors with more than 800 sequences encoded by about 4% of the human genome (1). GPCRs, which act as molecule sensors on the cell surface, lead to signal transduction by activation and/or inhibition of various intracellular signaling pathways leading to final cellular responses (2). Historically, the first structure determination of a GPCR was that of bovine rhodopsin, solved by Palczewski (3). Nearly 10 years later, the first structure of a human GPCR, the β_2_-adrenergic receptor (βAR), was determined by the group of Rasmussen et al. (4). In 2012, Lefkowitz and Kobilka were awarded with the Nobel Prize in Chemistry “for studies of G-protein-coupled receptors” (5). All GPCRs, also named seven-transmembrane receptor or 7-TM receptors, consist of seven integral α-helices transmembrane domains (H1 to H7) delineating extracellular domains (N-terminal domain and extracellular loops) classically involved in the ligand recognition and intracellular domains (C-terminal domain and intracellular loops) involved in the receptor regulation and signal transduction (6). An eighth α-helix (H8), which would be involved in Gβ/γ binding, has been identified through structural studies of GPCRs (3).

The nature of ligands interacting with GPCRs is characterized by a great diversity, including light, ions, amines, lipids, peptides, proteases, small, and large proteins having multiple properties as neurotransmitters, hormones, pheromones, and odors among others (7). The binding of these various ligands to GPCRs induces a structural conformational change and leads to the activation of G proteins (transducin, Gs, G_i/o_, G_q/11_, and G_12/13_). Two major signal transduction pathways that have been associated to GPCRs are the cAMP signal pathway through the adenylyl cyclase effector and the phosphatidylinositol signal pathway through the phospholipase C effector (8). In parallel to its role as a negative regulator of the α subunit, the dissociated Gβ/γ has the ability to modulate signaling pathway cascades including, among others, the regulation of ion channels, the inhibition or activation of adenylyl cyclase, the inhibition of the phosphinositide-3 kinase (PI3K) or the activation of GPCR kinases (βARK) (9).

GPCRs are classified into 6 groups according to IUPHAR nomenclature: rhodopsin-like (class A), secretin-like (class B), metabotropic glutamate (class C), fungal mating pheromone (class D), cyclic AMP receptors (class E), and frizzled/smoothened (class F). This large family of receptors is widely expressed in eukaryotes from yeast to human, and has an essential role in physiological processes, including homeostasis, hormone secretion, neurotransmission, cell differentiation, immunity regulation, vision, metabolism, muscle contraction, olfaction, pain, and many more (10). Related to the large involvement of GPCRs in human physiopathological conditions, these receptors play a major role in inflammatory diseases either by exacerbating and/or inhibiting inflammation (11). Naturally, our intent is not to describe all actions of the multitude of GPCRs in inflammatory contexts, but to outline some of their implications.

GPCRs are able to act directly on immune cells but also on non-immune cells present in specific tissues and organs (12). Among their major actions, they mediate cell migration, phagocyte activation, degranulation, the production of ROS (reactive oxygen species), vascular endothelial permeability and inflammatory nociception (11). Besides these actions, GPCRs are able to regulate inflammatory gene expression (13). GPCR-ligand binding leads to the modulation of transcription factors involved in inflammatory signaling cascades, such as CREB, ERKs, NFAT, c-Jun, STAT3, and NFκB among others (11). GPCRs have been involved in inflammatory diseases such as rheumatoid arthritis (14), sepsis (15), inflammatory bowel diseases (IBD) (16), pancreatitis (17), multiple sclerosis (18), chronic obstructive pulmonary disease (19), renal inflammation (20), and metabolic syndrome involved in obesity and diabetes (21). In that respect, the crosstalk between the actors of inflammation and GPCRs has led to consider these receptors as very promising targets with potential therapeutic applications in inflammatory pathologies. Among the 800 members of the GPCR family, orexin receptors represent an archetype of a putative target for the treatment of chronic inflammatory diseases (22).

**Figure 1:**
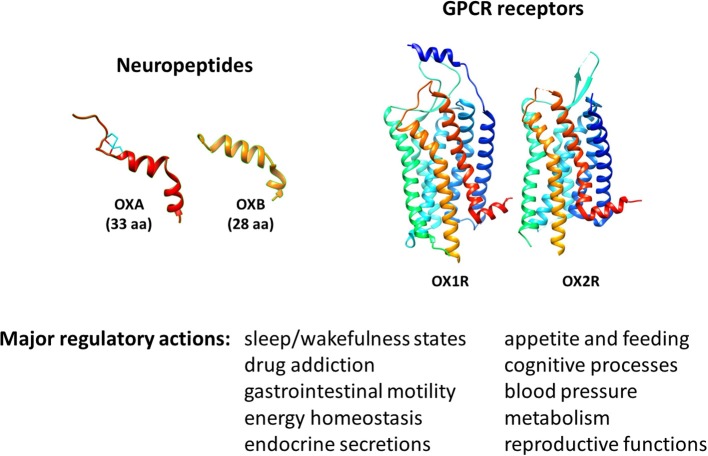
Molecular 3D representation and biological roles of orexins/OXRs system.

Orexins, also known as hypocretins, comprise two neuropeptides isoforms of 33 and 28 aminoacids, orexin A (OXA/hypocretin-1) and orexin B (OXB/hypocretin-2), respectively (Figure 1). They are encoded by a common precursor polypeptide named prepro-orexin (23). Originally discovered in the hypothalamus in the late nineties, lateral hypothalamic orexin neurons project, and release those peptides widely throughout the central nervous system (CNS) (24, 25). They were initially identified by reverse pharmacology as the endogenous ligands for two orphan GPCR subtypes belonging to the class A family, orexin receptor 1 and 2 (OX1R (Hcrt-1) and OX2R (Hcrt-2), respectively) (23, 25) (Figure 1). Signaling pathways that have been associated to orexin receptors are phospholipase A2, C and D, diacylglycerol lipase, Ca^2+^, and adenylyl cyclase cascades (26).

The major biological action of orexins is the regulation of sleep/wakefulness state (24, 27) (Figure 1). Related to this action, one major pathology associated to a deficit of orexin production is narcolepsy with cataplexy, referred to as type 1 narcolepsy (T1N). T1N is characterized by a severe dysregulation of the sleep/wakefulness cycles (28, 29). Accordingly, many academic and pharmaceutical laboratories have developed orexin receptor-targeting molecules, in particular antagonists, to treat insomnia (30, 31). These antagonists have been classified into two types depending on their ability to act on one or both orexin receptors: single orexin-receptor antagonists (SORAs) and dual orexin-receptor antagonists (DORAs). Furthermore, SORAs have been subdivided into two subclasses according to their receptor specificity, SORA1 (such as compound 56) and SORA2 (such as JNJ-42847922), targeting OX1R or OX2R, respectively (32, 33). Recently, the DORA molecule suvorexant (MK-4305) has been approved by the U.S. Food & Drug Administration (FDA) for the treatment of insomnia (34). In addition to their ability to modulate sleep and arousal states, these neuropeptides regulate appetite and feeding, gastrointestinal mobility, energy balance and metabolism, but also play a role in cognitive processes (35–40). Thus, multiple studies have highlighted the therapeutic potential of targeting the orexin system, not only in sleep, cognitive [i.e., Alzheimer's disease (AD)] and metabolic (i.e., obesity) disorders (41–45), but also in ischemic and oxidative stress events (46, 47) and in cancer (48, 49).

In addition to their actions in the CNS, these neuropeptides also play a role in various peripheral organs where they regulate appetite, feeding, gastrointestinal mobility, energy balance, metabolism, blood pressure, neuroendocrine and reproductive functions (36–41, 50) (Figure 1). In parallel, the expression of orexins in peripheral tissues has been investigated using immunochemistry and RT-PCR strategies which detected mainly the prepro-orexin precursor. Despite a large variability in terms of expression levels, orexins have been detected in adrenal glands (51), adipose tissues (51), kidney (52), colon (53), pancreas (52), and reproductive organs including testis (54, 55) and prostate (56). In parallel, orexin receptors are also expressed in peripheral tissues including the gastrointestinal tract, adrenal gland, endocrine pancreas, reproductive system, and adipose tissues (50, 57). In these tissues, a paracrine action of orexins is possible. In fact, the circulating level of orexins in blood in healthy individuals is very low [Range of 2 to 45 pM, representing 1,000 times less than the IC_50_ of receptors (58–60)]. Although the precise source of orexins in disease conditions remains to be elucidated, the abnormal expression of orexin receptors in certain human pathologies has been demonstrated and may lead to new therapeutic targets. In this sense, the ectopic expression of OX1R in human IBD and digestive cancers has been shown, and the administration of exogenous OXA led to a protective effect of orexins in animal models of these pathologies (22).

The existence of a bidirectional crosstalk between the nervous and immune systems has been revealed in the last decades. In this context, the present review will attempt to highlight the impact of the administration of exogenous orexins in the central nervous (i.e., neuroprotective properties) and immune (i.e., anti-inflammatory properties) systems in physiological and pathophysiological conditions including neuroinflammation, intestinal bowel diseases and systemic inflammation.

## Orexins and Neuroinflammation

In the CNS, the established relationship between neurons, microglia, and glial cells is highly dynamic and responsive to the diversity of environmental stimuli. For example, in response to injury, infection or disease, the cellular microenvironment of the CNS produces inflammatory mediators including cytokines, chemokines, adhesion molecules, prostaglandins, and free radicals. Those mediators stimulate the recruitment of additional immune cells as well as the activity of astrocytes and microglia. Particularly, in the healthy brain, microglia, resident macrophage-type immune cells of the CNS that share many characteristics with macrophages, are vital to preserve neuronal health (i.e., to promote formation and elimination of synapses) by maintaining a friendly CNS microenvironment. Indeed, microglial cells are capable of adopting appropriate phenotypic responses (i.e., inflammatory and activated vs. anti-inflammatory and resting) according to the type of stimuli. This immune reactivity of the CNS is beneficial and has to be under tight control to efficiently recover physiological homeostasis; however, long-term and dysregulated neuroinflammation, which is generally accompanied by a chronic inflammatory phenotype of microglia, can trigger deleterious effects on the CNS (i.e., subsequent and progressive neuronal loss). Thus, neuroinflammation is a key mechanism contributing to the progression and exacerbation of neurodegenerative and/or inflammatory diseases of the nervous system.

This concept gained even more credence with the discovery of neuropeptides exerting both neuroprotective and immunomodulatory actions, and becoming an emerging group of biological agents with a great potential for the treatment of immune-mediated CNS disorders such as narcolepsy, metabolic disorders, Alzheimer's disease, and multiple sclerosis. One potential candidate is the orexin system. Indeed, an increasing amount of evidence suggest a novel involvement of the orexin/receptor system in the immune and nervous systems. Particularly, as it will be discussed below, one of the two orexins, OXA, exhibits via activation of orexin receptors, neuroprotective, and immuno-regulatory actions, and thus its administration may be beneficial in the aforementioned diseases.

## Orexins Neuroprotective Actions

Recent studies have reported that the hypothalamic neuropeptide OXA (OXA, hypocretin 1) may exert an important role in neuroprotection, in part by reducing apoptosis and inflammation (47, 61, 62). Hence, using orexin/ataxin-3 (O/A3) mice, a transgenic mouse model of neurodegeneration, orexin loss has been linked to neurodegeneration, memory and cognitive deficits, and neuroinflammation (63). Supporting a role for endogenous orexin in neurodegenerative/inflammatory brain pathology, orexin expression was found to be elevated in lesioned CNS areas in murine controlled cortical impact (CCI) and transient common carotid artery occlusion (tCCAO), models of traumatic brain injury and cerebral ischemia, respectively (64, 65). In these studies, the cellular localization of orexin receptors was further investigated by immunofluorescence. Although orexin receptors expression is known to be neuronal in healthy brain tissue, expression by glial cells was also reported in these models. For example, OX1R receptor was found to be upregulated in microglia after CCI (64). Moreover, astrocytes and oligodendrocytes were found to express OX1R after tCCAO (65). Although evidence in human pathology is missing, these studies may suggest a potential action of orexin not only on neurons, but also on glial cells.

Data indicate that orexin-induced neuroprotection could rely upon microglial modulation (62, 66). Microglia behave like a sentry capable of efficiently react to endogenous signaling in order to initiate proper neuroinflammatory responses through dynamic transitioning between neurotoxic pro-inflammatory (M1) and neuroprotective (M2) phenotypes. For example, following cerebral ischemic events, microglia can adopt two phenotypes: first an activated neuroprotective M2 phenotype together with the reduction of oxygen levels, and then switch to a pro-inflammatory M1 phenotype, provoking cell death (67). While inflammation is a required normal immune response, chronic M1 pro-inflammatory activation can be detrimental and contributes to subsequent neuronal dysfunction and damage (68). In this context, numerous evidences shed the light on the orexins/receptors system involvement. Indeed, *in vivo*, OXA exhibited potent neuroprotective actions in several models of rodent focal cerebral ischemia, reducing infarct size (46, 62, 69). This set of data implies a mechanism driven by microglia (46, 62, 69).

Several *in vitro* studies have demonstrated that OXA promotes both neuronal survival and neuronal protection from death caused by oxidative and hypoxic stress. For example, orexins A and B were capable of efficiently protecting primary rat cortical neurons against cobalt-induced oxidative stress (70). Using SH-SY5Y human neuroblastoma cell line, an *in vitro* cellular model of dopaminergic neurons in Parkinson's disease, other investigators have shown that OXA elicited neuroprotective actions (i.e., anti-apoptotic and antioxidant effects which are mediated by the PKC and PI3K signaling pathways) against MPP(+) and 6-OHDA-induced neurotoxicity (71–73). These *in vitro* results might be relevant in light of MS pathogenesis. Indeed, accumulating evidence suggests that oxidative stress, at least in part, contributes to MS pathophysiological processes such as demyelination, axonal damage and neuronal death. In another study, a microarray analysis of neuronal differentiated SH-SY5Y cells treated with OXA revealed the upregulation of somatostatin receptors, vasoactive intestinal peptide (*VIP*), endothelin-1 (*EDN1*), and members of the NF-κB pathway, all of which contribute to neuroprotection (74).

### Orexins Immuno-Regulatory Properties

In addition to its effects in the nervous system, several studies have shown that OXA can act *in vivo* as an anti-inflammatory neuropeptide, further supporting its therapeutic potential in neurodegenerative and/or inflammatory disorders. In a rat model of ischemia reperfusion-induced gastric damage, the infusion of OXA: (1) dramatically reduced gastric damage by diminishing the production of reactive oxygen species (ROS) and (2) reduced myeloperoxidase activity in the gastric tissue, suggesting a decrease in polymorphonuclear infiltration and/or activity (75). Later on, using a murine focal cerebral ischemia model, another group demonstrated that the extent of brain lesions were attenuated by the endogenous orexin system, an effect associated with reduced inflammation (i.e., decrease of IL-6 and TNFα levels) (76). More recently, peripheral administration of orexin reduced the levels of proinflammatory mediators (i.e., cytokines and chemokines) and improved the survival of mice in the model of lipopolysaccharide (LPS)-induced endotoxin shock (77). In addition, exposure to LPS down-regulated orexin signaling, supporting the contribution of orexins during an inflammatory event (78). Interestingly, this study demonstrated that peripherally administered OXA was able to cross the blood brain barrier (BBB) under endotoxin shock conditions and acted directly to reduce inflammation in the CNS. This evidence strongly suggests that the orexinergic system can exert its beneficial immuno-regulatory functions not only in inflammatory, but also in immune-driven neurodegenerative diseases.

Despite the scarcity of data regarding the expression of orexin receptors in immune cells, we found that OX1R and OX2R receptors are expressed in murine central and peripheral immune cell tissues, and particularly in sorted T (CD4^+^ and CD8^+^) and myeloid (CD11b^+^) cells (79). We have also described the expression of OX1R in murine colonic *lamina propria* immune cells (80).

The cellular and molecular mechanisms by which OXA exerts its anti-inflammatory actions in those models have been poorly investigated, with mostly *in vitro* studies performed. Indeed a direct effect of orexin signaling on microglial cell lines has been shown (62, 66). In normal circumstances, the potent pro-inflammatory agonist lipopolysaccharide (LPS) increases TNF-α production in microglial cell line BV-2 as well as OX1R expression. Interestingly, Xiong et al. reported that a pre-treatment with OXA of the BV-2 cells prior to LPS exposure led to a reduction of TNF-α (62). Although this might suggest an action on innate immune cell mechanisms, the limitation of this work is its *in vitro* nature. Further studies would be required to demonstrate the relevance of this data as a mechanism for orexin immunoregulatory properties *in vivo*.

Overall, these recent findings suggest a therapeutic potential of OXA in inflammatory diseases of the CNS.

## Orexins in Disease

### Narcolepsy

Type 1 narcolepsy (T1N) is a rare but severe chronic neurological sleep disorder (81). Its main symptoms are an excessive daytime sleepiness, cataplexy (sudden loss of muscle tone), fragmented night time sleep with episodes of sleep paralysis and hallucinations (81). T1N is triggered by a selective and almost complete destruction of orexinergic neurons in the lateral hypothalamus (82, 83). Numerous evidence obtained from risk factor studies (i.e., genetic and environmental) and serologic data, suggest that T1N pathogenesis is an autoimmune-based process. A high association of the disease incidence has been found with certain human leukocyte antigen (HLA) class I alleles (i.e., HLA-DQB1^*^06:02 allele) (84–86), with polymorphisms in the α chain locus of the T-cell receptor (TCR) (87, 88), with the presence of autoantibodies against different CNS antigenic targets identified in the serum and cerebrospinal fluid (CSF) (89–91) and with the vaccination campaigns (i.e., Pandemrix vaccine) against pandemic H1N1 influenza virus (92–94). Even if it has to be confirmed, molecular mimicry has been proposed as a pathophysiological mechanism of the disease (91, 95).

In order to study the autoimmune mechanisms involved in the development of narcolepsy and particularly to discover the effector immune cells responsible for the selective orexin-secreting neuron destruction, a novel mouse model of narcolepsy has been generated (96). Mice were designed to express a “neo-self-antigen” [i.e., hemagglutinin (HA)] specifically in hypothalamic orexin-expressing neurons (named Orex-HA). To induce the disease, they were then adoptively transferred with effector neo-self-antigen-specific T cells either CD4^+^ Th1 or cytotoxic CD8^+^ (CTLs). Both HA-specific T cells were able to infiltrate the hypothalamus and cause local inflammation. However, only CTLs were capable of leading to a narcoleptic-like phenotype mimicking human T1N clinical manifestations such as cataplexy and sleep attacks. The latter phenotype was accompanied with a selective and drastic destruction of orexin^+^ neurons due to a direct and antigen-dependent CTL-mediated cytotoxicity. This work thus emphasizes that narcolepsy pathogenesis is strongly mediated by the immune system (i.e., CTLs play a central effector role) and suggests that novel therapeutic strategies including OXA should trigger the protection of orexin-secreting neurons.

Studies using orexin receptor transgenic mice have suggested a major role for OX2R in narcolepsy. Indeed, narcolepsy in dogs has been associated with a deficiency of the OX2R (29), and narcolepsy-cataplexy symptoms have been observed in OX2R- and not in OX1R- deficient mice (97, 98). Moreover, wakefulness is inhibited only by OX2R and dual orexin receptor antagonists, but not by selective OX1R antagonists (99). Recently, it was shown that peripheral administration of a potent non-peptidic OX2R agonist, YNT-185, significantly ameliorated narcolepsy symptoms in mice (100). This study supports a therapeutic use for orexin receptor agonists (in particular OX2R agonists) as a therapy in narcolepsy.

### High Fat Diet (HFD)-Induced Obesity

Given the fact that: (1) the orexin system efficiently controls appetite and feeding as well as the energy balance and metabolism and (2) OXA exhibits potent neuroprotective function, for example by attenuating oxidative stress-induced cell death, another team was interested in deciphering how the dynamic orexin-microglia dialogue might interfere with brain health to induce obesity through high fat diet in saturated fatty acids (SFA) [i.e., palmitic acid (PA, C16:0)] exposure (66). Chronic dietary intake enriched in PA contributes to hypothalamic neurodegeneration (i.e., neuronal cell death and apoptosis) in part through earlier onset of increased oxidative stress, overproduction of ROS, insulin resistance, and hippocampal neuroinflammation (i.e., release of circulating proinflammatory cytokines from microglial cells) (63, 101–105). Additionally, HFD-induced ROS leads to an impairment in hypothalamic gene expression profiles linked to obesity pathogenesis including downregulation of the neuronal anti-apoptotic protein B cell lymphoma 2 (Bcl-2), but upregulation of the pro-apoptotic protein B cell lymphoma 2 associated X protein (Bax) (106, 107).

Using the immortalized murine BV-2 microglial cell line, authors have shown that PA treatment: (1) increases OX1R gene expression but not OX2R and (2) causes the BV-2 cell line to shift toward a pro-inflammatory M1 state (66). In parallel, other teams demonstrated that PA diet activates microglia to an M1 phenotype, resulting in the release of pro-inflammatory cytokines such as TNF-α and IL-6, under either a NFκB- or a toll like receptor 4 (TLR-4)-dependent pathway (66, 103, 108, 109). Further, microglial activation by SFA *via* TLR-4 contributes to neuronal cell death (108). However, OXA efficiently blocked the harmful effects of PA. Indeed, OXA is capable of promoting a neuroprotective anti-inflammatory M2-like microglial phenotype at the expense of the PA-induced neurotoxic pro-inflammatory microglial M1 phenotype. This was characterized by increased expression of the M2 microglial marker arginase-1, while inhibiting the production of pro-inflammatory TNFα, IL-6 and inducible nitric oxide synthase (iNOS) mediators (66). In addition, using an immortalized murine hypothalamic neuronal cell line (named as mHypoA-1/2), Duffy et al. showed that OXA protects hypothalamic neurons against PA-induced hypothalamic microglial dysregulation (110). This beneficial effect was accompanied with: (1) diminished caspase-3/7 apoptosis, stabilization of Bcl-2 gene expression, and subsequent decrease of Bax/Bcl-2 gene expression ratio, (2) inhibition of ROS production, and (3) a reversion of PA-induced changes in intracellular metabolism, basal/maximum respiration, ATP production and reserve capacity (110). These data support the concept that OXA can efficiently block the actions of PA and may act as a potent immuno-regulator of M1/M2 phenotype microglia, reducing pro-inflammatory cytokines and increasing anti-inflammatory cytokines to promote a beneficial neuronal microenvironment.

### Alzheimer's Disease (AD)

Alzheimer's disease is primarily characterized by the loss of pyramidal neurons and synapses in the cerebral cortex as well as in some subcortical regions such as the hippocampus. This event results in general in brain atrophy as well as expanded ventricular volume. Alongside intracellular aggregates of hyper phosphorylated tau proteins and extracellular deposits of amyloid-β (Aβ) aggregates (111), both clinical and preclinical studies have provided recent data clearly determining that AD is a multistep disorder in which chronic and uncontrolled neuroinflammatory processes play an important role for its development. Initially, to preserve healthy brain function, inflammatory responses against Aβ deposits from microglia and astrocytes coordinate efficient phagocytic removal and enzymatic breakdown of amyloid peptides, respectively. However, AD patients present excessive tau protein and Aβ deposition that overcomes physiological clearance, resulting in continued microglial stimulation. The latter significantly leads to an overproduction of pro-inflammatory cytokines which foster dysregulated neurodegeneration (i.e., death of otherwise healthy proximal neurons) in the brain microenvironment (112, 113). In addition, cellular debris and damage-associated molecular patterns from these degenerating neurons can further enhance the stimulation of microglia and the production of inflammatory mediators. It has been shown that diet factors, such as PA, potentiate the risk of not only developing obesity but also cognitive disorders such as AD. In this regard, as mentioned before, several teams have demonstrated the beneficial effects of OXA by antagonizing the proinflammatory actions of PA diet (62, 63, 66, 110). The pathogenic role of excessive inflammation in AD suggests that an antiinflammatory treatment may exert beneficial actions in the disease.

Sleep is critical for physiological brain function allowing the clearance of neurotoxic waste products such as Aβ (114) and stimulates synapse formation and maintenance during the learning process (115, 116). In contrast, sleep deprivation leads to inflammation, reactive glia response, reduced Aβ clearance ability (114, 117) and subsequently strongly increases its levels in the hippocampus and cortex (41) in AD-relevant mouse models. In this sense, increasing evidence in mice and in human suggests that sleep disruption may exacerbate the progression of Alzheimer's neuropathology and cognitive deterioration including memory (118–121). In this context, Duncan et al. tested whether a chronic administration of a dual orexin receptor antagonist (DORA) would favor sleep enhancement and attenuate the development of AD by reducing neuropathology, neuroinflammation, and cognitive deficits. For this purpose, an AD-relevant mouse preclinical model (i.e., 5XFAD mice) which exhibits AD-like features (i.e., sleep disruptions, neuropathology, neuroinflammation, and cognitive deficits including spatial memory) was chosen for the study. In 5XFAD mice, DORA significantly increased light-phase sleep and restored natural sleep patterns (122). Nevertheless, it did not impact neuropathological and neuroinflammatory features of the disease with similar Aβ levels and plaque density in comparison with untreated-DORA mice. 5XFAD mice did not exhibit cognitive deficits in this study. Therefore, the authors could not evaluate whether or not DORA-induced increased sleep improved cognitive functions (122). This set of findings suggests that OXA antagonist analogs (DORA) may be used to improve sleep pattern in AD patients, but its impact on neuroinflammation remains unknown.

Thus, we might speculate that whereas OXA agonists might decrease inflammation (i.e., in the case of high fat diet associated pathology) and potentially AD, antagonist molecules might be beneficial by improving sleep patterns in AD patients with sleep deficits. Further research is needed to determine the best orexin-based therapy in AD.

### Multiple Sclerosis (MS)

Multiple sclerosis is a chronic demyelinating disease of the central nervous system. MS is initially characterized by episodes of acute symptoms, followed by partial or complete recovery (relapsing-remitting MS), until remissions no longer occur and disability continuously progresses (progressive MS). Despite its complex pathogenesis, it is established that chronic inflammation in the spinal cord and brain is driven by a Th1/Th17 autoimmune component of the disease. This is characterized by exacerbated neurodegeneration and failure of central nervous system repair mechanisms. Thus, most of MS therapies are immunomodulatory. However, current treatments are only partially effective at the earliest phases of the pathology slowing its progression of disability and also reducing its severity and incidence of exacerbations with somehow important side effects, and have no major impact on its progressive phase (123–125). In addition to inflammation, axonal and neuronal pathologies are central components during MS.

The aforementioned evidences suggest that OXA may present potent therapeutic properties for MS by acting on both inflammatory and neurodegenerative components of the disease. An upregulation of hypothalamic orexin receptor mRNA expression has been described upon experimental autoimmune encephalomyelitis (EAE, a widely used MS mouse model) (126). The same team has shown that intracerebroventricular (ICV) delivery of OXA starting before disease onset, attenuated the clinical score of EAE (127). Nevertheless, this brief study did not address whether or not OXA was able to dampen key components in MS pathogenesis, such as Th1 and Th17 encephalitogenic responses and neurodegeneration. Compared to ICV administration, peripheral administration of OXA might be more interesting from a therapeutic standpoint. Whereas, IP-delivered OXA might easily reach immune organs, a desired local action at the level of the CNS, notably to provide neuroprotection, would require that this neuropeptide crosses the BBB. There is not much information in this sense, but one study demonstrated that intravenously delivered OXA was capable of crossing the BBB from the blood by simple diffusion (128). Furthermore, as mentioned before, peripherally (IP) administered OXA was capable of crossing the BBB and reach the CNS in a study of LPS-induced systemic inflammation (77). This suggests that OXA administered peripherally might act both at CNS and peripheral levels. In order to respond to these issues in MS mouse models, we investigated the curative potential of peripheral OXA administration in the clinical development of ongoing established chronic myelin oligodendrocyte glycoprotein 35-55 (MOG_35−55_)-induced EAE (a mouse model for progressive MS). Moreover, we studied the impact of this treatment on inflammatory and neurodegeneration processes that underlie the pathogenesis of EAE. We found that an intraperitoneal OXA administration to mice undergoing established chronic MOG_35−55_-induced EAE had a striking curative effect by alleviating the clinical symptoms and histopathological features of the disease. This was associated to a global reduction of the inflammatory response in the CNS, including a decrease of immune cell infiltration (i.e., CD4^+^ T cells) and the expression of immune cell mediators (chemokines such as MCP-1/CCL2 and IP-10/CXCL10, and cytokines such as IFN-γ, IL-17, TNF-α, IL-10, and TGFβ) (79). In parallel, OXA diminished demyelination, astrogliosis and microglial activation. The immunomodulatory effects of OXA were not observed in the periphery, since OXA failed to inhibit *in vitro* draining lymph node cell responses to MOG_35−55_ (proliferation and Th1/Th17 cytokine production) (79). Overall, this set of results provided the proof-of-concept that peripheral administration with OXA may be beneficial in MS.

### IBD

Intestinal bowel diseases (IBD) encompassing Crohn's disease (CD) and ulcerative colitis (UC) are characterized by chronic inflammation of the intestinal mucosa (129). UC is a crippling disease characterized by relapsing-remitting cycles affecting exclusively the mucosa of the colon and rectum following a distal to proximal inflammatory gradient (130). This inflammatory disease was described during the acute phase by the change of mucosal structure resulting of an alteration of mucus-secreting goblet cells, crypt distortion, and crypt abscesses induced by an immune cell infiltration trough the epithelium (130). In this respect, the presence of these lesions might, at least in the longer term, evolve toward dysplasia and colorectal cancer (CRC) (131). The major symptoms are abdominal pain, persistent diarrhea including bloody stools, weight loss, and large fatigue (130). The incidence of this pathology is about 300 per 100,000 in the USA with a general prevalence of IBD of 0.3 % in North America, Oceania and Europe (132). At date, the exact cause of UC remains mainly unknown. UC is a multiple pathogenic disease involving various factors, including genetic susceptibility, environmental impact, dysbiosis, dysregulation of innate and adaptive immune response, inflammasome signaling pathway, regulatory RNAs and endoplasmic reticulum (ER) cellular stress (133).

**Figure 2:**
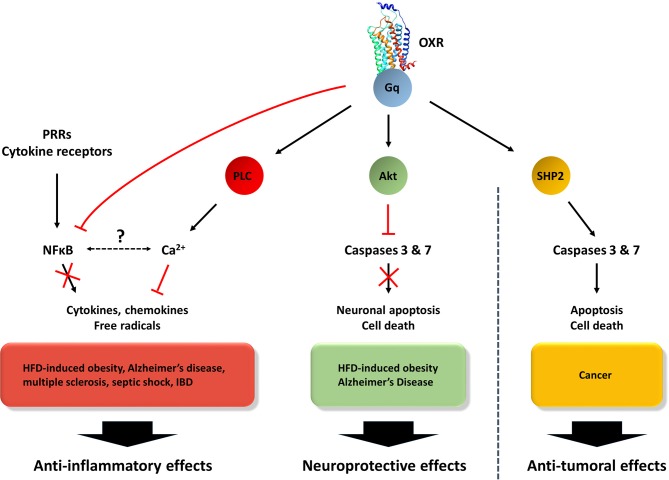
Orexins/OXR system-modulated signaling patways involved in anti-inflammatory, neuroprotective and anti-tumoral effects. The orexin/receptors system may trigger: (1) anti-inflammatory functions through the inhibition of NFκB and the activation of PLC/Ca^2+^ pathways, (2) in the CNS, neuroprotective actions via the inhibition of caspases 3/7 by Akt pathway and (3) in the context of cancer, anti-tumoral effects through the activation of caspases 3/7 by SHP2 signaling pathway (60).

Currently, the treatment of UC is dependent on the severity of disease. The first line of treatment involves anti-inflammatory drugs, including 5-aminosalicylates and corticosteroids (134). The use of immune system suppressors such as azathioprine, methotrexate, cyclosporine, anti-TNFα (Infliximab) and anti-integrin/α4β7 (Vedolizumab) is also prescribed alone or in combination (134). Unfortunately, the failure of medication, a significant degradation in the quality of life and/or severe flare-ups including acute severe colitis, perforation, uncontrollable bleeding and risk of cancer leads to perform surgery consisting of total colectomy (134). In this context, the identification of new targets represents a major goal in the treatment of this pathology. GPCRs may constitute these innovative new targets. Indeed, most of those receptors are potential targets in colitis such as chemokine receptors (135), cannabinoid receptors (136), histamine receptors (137), and neuropeptide receptors (138). Recently, it was shown that OX1R was expressed in inflamed mucosa from patients having UC (80), but not in normal mucosa (139). It should be emphasized that an UC rat model reproducing chronic mucosal inflammation by injection of adjuvant mixture containing proteins from UC patients, revealed an upregulation of OXA in colon (140). Moreover, an epidemiologic analysis from narcoleptic patients indicated a higher prevalence of immunopathological diseases, including among others Crohn's disease and ulcerative colitis (141). In the classical DSS-induced acute colitis mouse model, OX1R was found to be highly expressed in inflamed colon mucosa whereas the receptor was not expressed in normal mucosa (80). Three intraperitoneal injections of OxA by week in this animal model resulted in an anti-inflammatory effect characterized by the restoration of the intestinal barrier and the inhibition of TNFα, IL-1α, IL-1β, IL-6, IFNγ, IL-17 cytokines and the MCP-1 chemokine in the colon mucosa (80). More recently, Tunisi et al. have demonstrated that OxA prevents the disruption of the intestinal barrier induced by LPS in Caco-2 cells and *in vivo* (142). The use of a genetically modified murine model where the IL-10 and NAPDH Oxydase 1 (NOX1) genes were invalidated, which mimics the chronic phase of human UC (80), has demonstrated the same anti-inflammatory effect of OXA. Moreover, these OXA-induced anti-inflammatory effects were specific because they were reverted by: (1) the SORA molecule SB-408124 which is an OX1R specific antagonist (80) and (2) the use of an OX1R^−/−^ mouse model in which UC was induced by DSS (80). The anti-inflammatory effect of OXA was mainly mediated by the activation of intracellular calcium releasing signaling pathway and by the inhibition of the NFκB activation (Figure 2) (80). In this study, OX1R was expressed by T lymphocytes and its activation by OXA led to an inhibition of pro-inflammatory cytokines (80).

### Digestive Cancers

Considering that chronic inflammation encompassing IBD, pancreatitis, hepatic fibrosis and metabolic syndrome constitute a high-risk factor to develop cancers (131), the role of orexins in inflammation represents a major question. In 2011, our group had demonstrated that OX1R but not OX2R was highly expressed in colon cancer cell lines and colorectal tumors from patients (139). It should be noted that: (1) no detection of prepro-orexin was observed in normal and tumoral colonic epithelia (139) and (2) OX1R was not detected in normal colon epithelium (22). Orexins treatment of digestive cancer cell lines derived from colon, pancreas and liver cancers (22, 139, 143) induced a strong cell death by apoptosis. The orexin-induced apoptosis was mediated by the phosphorylation of two tyrosine-based motifs (ITIM) present in the receptor sequence. This triggered successive signaling events (Figure 2), such as: the recruitment of the phosphotyrosine phosphatase SHP2, the phosphorylation of the p38 mitogen/stress-activated protein kinase and the translocation of the proapoptotic protein Bax in the mitochondria, leading to apoptosome formation and caspase (3 and 7) activation (144, 145). In a preclinical model, where cancer cells lines derived from colon, pancreas, liver and prostate cancers were sub-cutaneously xenografted, OXA treatment induced a strong reduction of tumor volume (22). This anti-tumoral effect of OXA was also observed in the patient-derived xenograft model (143). The expression of OX1R in digestive cancers had occurred at an early stage since the dysplastic cells present in colon polyps or pancreatic intraepithelial neoplasia (PanIN) lesions highly expressed OX1R (22, 143).

### Septic Shock

Sepsis is a systemic infection syndrome representing one of the most important causes of admission in the intensive care unit and potentially life threatening (146). Septic shock, which is associated to organ(s) dysfunction, is the culmination of sepsis through a continuum between infection to severe sepsis (147). It should be noted that the difference between sepsis and the systemic inflammatory syndrome (SIRS) is only related to the presence of one identifiable focus of infection in sepsis (147). Clinical signs associated with septic shock encompass fever, hypotension, tachycardia, oliguria, respiratory distress, skin marbling, confused thinking, and they can evolve to coma. Except for the intensive care associated with organ failure, including heart, kidney, respiratory tract, liver and brain, the treatment of septic shock consists of intravenous injection of empiric antibiotics, vasopressor medications, insulin and corticosteroids (148).

Although the pathological mechanisms involved in organ failure associated to septic shock are not completely understood, some candidate factors involved have been identified. An exacerbated secretion of inflammatory cytokines such as TNFα, IL-6, IL-1β, and MCP-1 has been described in sepsis (147). Associated with this cytokine storm, NFκB, which plays a central role in the induction of transcription pro-inflammatory genes, has been involved in septic shock (147). Indeed, the use of NFκB inhibitors as pyrrolidine dithiocarbamate and parthenolide in lipopolysaccharide (LPS)-induced septic shock murine models improved organ failure and hypotension (149). Cellular apoptosis process also plays a prominent role in septic shock. For example, T and B cell apoptosis has been reported in septic shock patients, leading to immunosuppression (147). Apoptosis of intestinal and lung epithelial cells has been also observed in autopsied patients (150). In addition, LPS, which is one of the major component of gram negative bacteria walls, is involved in septic shock. LPS interacts with the complex toll-like receptor 4 (TLR4)/myeloid differentiation factor 2 (MD-2). TLR4 is expressed in various cells such as macrophages, dendritic cells, adipocytes, enterocytes and mucosal cells, in which LPS induces cytokine and interferon secretion *via* NFκB activation (151).

Several GPCRs and their ligands have been involved in septic shock and/or in its treatment, including chemokine receptors (i.e., CCR2, CX3CR1, and CXCR1), neuropeptides (i.e., VIP, neuropeptide Y, ocytocin, vasopressin, neurotensin, orexins, substance P, and apelin), proteases [i.e., thrombin (PAR1 and PAR2)], lipid derivatives [i.e., N-arachidonylglycine (GPR18)] and amines (i.e., catecholamines, dopamine histamine, melatonin) (152–156).

The standard animal model used to study the role of GPCRs, particularly orexin receptors, in systemic inflammatory responses in the absence of infection, has been the endotoxemia model induced by LPS injection (157). In parallel, other models using either live bacteria administration or cecal ligation and puncture (CLP) which exposes the cecal content rich in bacteria into the peritoneal cavity have been used (157). In the early 2010s, Deutschman et al. using the CPL mouse model had demonstrated that the orexinergic activity was strongly reduced (158). This inhibition was associated with a reduction of respiratory, heart, temperature and arousal rates (158). Conversely, the intravenous injection of OXA reverted these clinical signs. Other reports indicate that LPS or TNFα (a major cytokine involved in septic shock) were also able to suppress orexin neuronal activity (159). More recently, the use of orexin-neuron ablated mouse model (OX/ataxin-3 transgenic mouse model) injected with LPS revealed a high mortality rate as compared to wild type mice (160). Moreover, the injection of LPS in wild type mice reduced OXA tissue content compared to untreated mice (160). Yanagisawa's group had clearly shown that the subcutaneous diffusion of OXA using an osmotic pump in LPS-induced endotoxin shock mice improve the survival of these mice (77). OXA ameliorated hypothermia and bradycardia associated to LPS-induced endotoxin shock, and reduced the secretion of TNFα, CCL3, IFNγ, IL-17, and IL-6 (77).

## Conclusions

**Figure 3:**
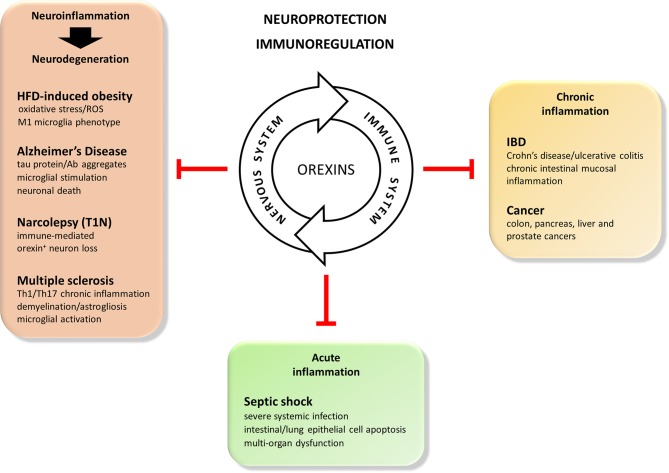
Beneficial actions of the orexins in immune mediated disorders. Orexin neuropeptides are known to efficiently regulate sleep and arousal states, appetite and feeding, gastrointestinal motility, metabolism, cognitive processes. More recently, considering the synergic and dynamic cross-talk between the nervous and immune systems, they also display potent neuroprotective and immunoregulatory features. Therefore, the orexin/receptors system may be consider as a potential therapeutical tool for the treatment of: (1) acute inflammatory-induced diseases such as septic shock, (2) chronic inflammatory inflammatory-induced diseases such as inflammatory bowl diseases (IBD) or cancer, and (3) immune-mediated neurodegenerative diseases of the central nervous system such as high fat diet (HFD)-induced obesity, Alzheimer's disease, narcolepsy (T1N), or multiple sclerosis.

Recent literature suggest that the orexin/receptor system can be added to the list of nervous system mediators exhibiting immunoregulatory properties. Overall, the *in vivo* and *in vitro* studies gathered here strongly indicate that, in addition to their conventional actions (Figure 1), orexins are neuropeptides with important neuroprotective and anti-inflammatory properties. This may expand their current interest as therapeutic agents from sleep disorders to neurodegenerative disorders with/without a neuroinflammatory component (i.e., HFD-induced obesity, Alzheimer's disease, narcolepsy and multiple sclerosis), acute inflammatory diseases (i.e., septic shock) and chronic inflammatory diseases (i.e., inflammatory bowel diseases and associated cancers) (Figure 3). Based on the data gathered in this review, Figure 2 summarizes potential molecular mechanisms leading to these effects.

## Author Contributions

AC, TV, PN, VG, CA, and YVT have actively participated in the writing of this manuscript.

### Conflict of Interest

The authors declare that the research was conducted in the absence of any commercial or financial relationships that could be construed as a potential conflict of interest.
